# Respiratory and intestinal trichobezoar in a female: An unusual case report and literature review

**DOI:** 10.1002/rcr2.1216

**Published:** 2023-09-18

**Authors:** Siavash Abedi, Masoud Aliyali, Amir‐Hassan Bordbari, Mostafa Soleymani, Zakaria Zakariaei, Mahdi Fakhar

**Affiliations:** ^1^ Pulmonary and Critical Care Division, Iranian National Registry Center for Lophomoniasis (INRCL), Imam Khomeini Hospital Mazandaran University of Medical Sciences Sari Iran; ^2^ Student Research Committee Faculty of Medicine, Mazandaran University of Medical Sciences Sari Iran; ^3^ Iranian National Registry Center for Lophomoniasis and Toxoplasmosis, Imam Khomeini Hospital Mazandaran University of Medical Sciences Sari Iran; ^4^ Toxicology and Forensic Medicine Division, Orthopedic Research Center, Imam Khomeini Hospital Mazandaran University of Medical Sciences Sari Iran

**Keywords:** bezoars, respiratory trichobezoar, trichobezoars, trichophagia, trichotillomania

## Abstract

Trichobezoars are hairballs in the gastrointestinal tract that usually develop due to the consumption of hair after pulling it. However, some rare case reports suggest that trichobezoar can also occur in the respiratory system. In this context, we present an unusual case of a 25‐year‐old woman who experienced dyspnea, productive cough, and leukocytosis. The patient was found to have a trichobezoar in her respiratory tract, accompanied by the presence of hair in her digestive tract.

## Graphical abstract

Trichobezoars are hairballs in the gastrointestinal tract that usually develop due to the consumption of hair after pulling it. We present an unusual case of a 25‐year‐old woman who experienced dyspnea, productive cough, and leukocytosis and was found to have a trichobezoar in her respiratory tract, accompanied by the presence of hair in her digestive tract.

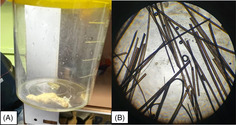

## INTRODUCTION

Trichobezoar is the accumulation of hair in the digestive system, which occurs due to the habit of pulling and consuming hair.^1^ The term ‘tricho’ originates from Greek, meaning hair, while ‘bezoar’ is derived from the Persian word ‘pādzahr’, translating to antidote, reflecting ancient beliefs about them.^2^ While other types of bezoars like phytobezoars (vegetable) and pharmacobezoars (undigested medication) exist, trichobezoar is the most frequently encountered form.^1^, ^3^

Although trichobezoars are commonly observed within the gastrointestinal (GI) tract, they can also occur in the respiratory system.^1^, ^4^ Patients with GI trichobezoars may present a diverse range of clinical complications, such as chronic abdominal pain, gastric outlet obstruction, nausea, vomiting, weight loss, malnutrition, food intolerance, diarrhoea and constipation.^2^ Moreover, respiratory trichobezoars have been linked to increasing dyspnea, snoring, hoarseness, and dysphagia.^1^, ^4^

In this report, we describe an uncommon case where hair was found in both respiratory and GI tracts.

## CASE PRESENTATION

A 25‐year‐old woman was referred to our tertiary hospital due to progressively worsening dyspnea and a productive cough that had lasted for 7 months. The patient reported experiencing shortness of breath even when resting, which worsened when lying down, along with sleep disturbances. Her sputum production was scant and malodorous, and she also complained of epigastric pain, nausea, vomiting after coughing, anorexia, and slight weight loss. About a month before being admitted to our hospital, the patient had been admitted to a local hospital with leukocytosis and eosinophilia (more than 2000/μL). While the patient had used salbutamol for a few weeks, she denied having any significant family or allergy history.

Upon admission, the patient presented with tachycardia, with a heart rate of 123 beats per minute, and a respiratory rate of 23 breaths per minute. Her oxygen saturation was 94% on room air. During the physical examination, generalized expiratory wheezing and coarse crackles were observed upon lung auscultation. A lung computerized tomography (CT) scan revealed a subpleural nodule in the right middle lobe and ground‐glass opacity in the left lower lobe. The patient's laboratory test results showed a white blood cell count of 34,100/mm^3^, with 10% lymphocytes, 88% neutrophils, and 2% eosinophils. Additionally, her erythrocyte sedimentation rate (ESR) was 20 mm/h, and her C‐reactive protein (CRP) was 14.9 mg/L (Table 1). Other laboratory tests were within normal limits. Stool examination revealed 5–6 red blood cells and 1–2 white blood cells, and was positive for occult blood. The patient underwent a colonoscopy which was unremarkable, though hair filaments were noted. Although bronchoscopy was considered as a therapeutic and or diagnostic tool, but it was not used due to lack of bronchial obstruction in imaging.

**TABLE 1:** Laboratory test results.

Laboratory test	Result	Reference values
WBC	34.1 × 10^3^/mm^3^	4 × 10^3^–10 × 10^3^/mm^3^
Lymphocytes	10%	
Neutrophils	88%	
Eosinophils	2%	
Haemoglobin	12.6 g/dL	11.7–16 g/dL
Platelet	299 × 10^3^/mm^3^	130–400 × 10^3^/mm^3^
CRP	14.9 mg/dL	Up to 6 mg/dL
ESR	20 mm/h	0–20 mm/h
Urea	23 mg/dL	13–43
Creatinine	0.7 mg/dL	0.6–1.3
Na	140 mEq/L	135–145 mEq/L
K	3 mEq/L	3.5–5.5 mEq/L
Stool Colour	Brown	
Consistency	Formed	
Stool RBC	5–6	
Stool WBC	1–2	
Occult Blood	Positive	

Abbreviations: CRP, C‐reactive protein, ESR, erythrocyte sedimentation rate; WBC, white blood cells.

**FIGURE 1:**
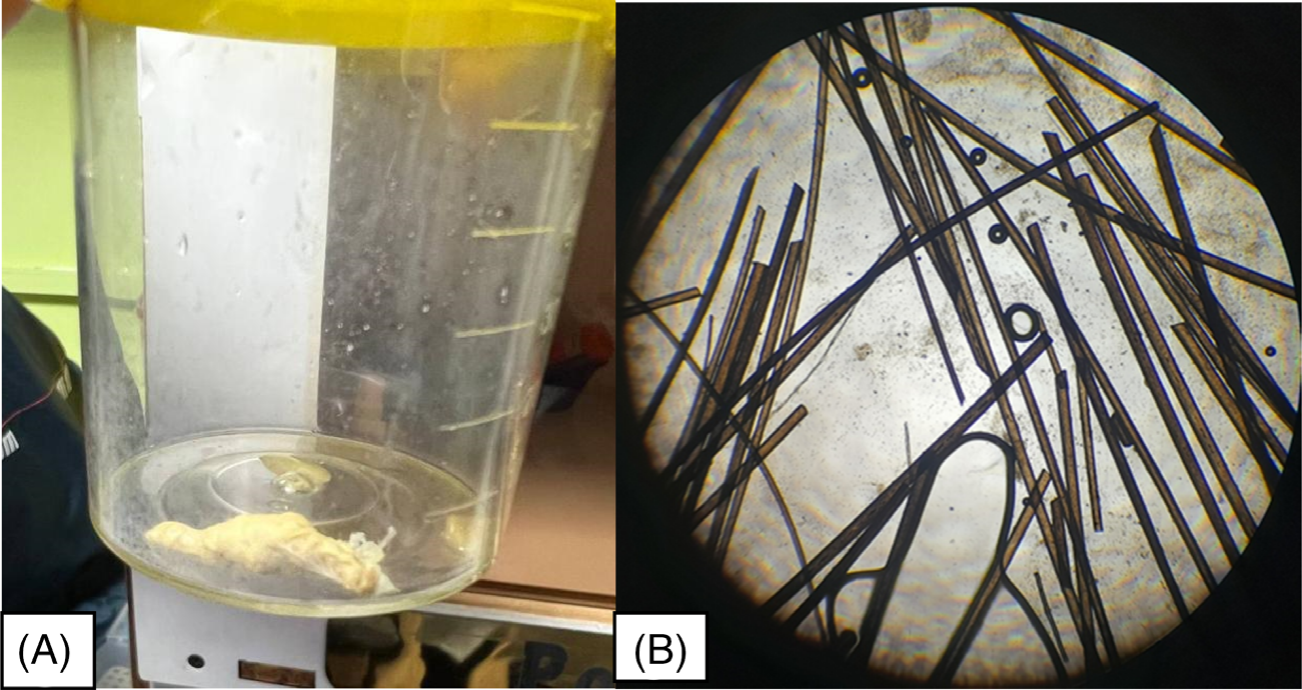
(A) white compact sputum sample containing hair; (B) light microscopic image showing hair filaments within the sputum specimen.

The patient was started on 4 mg intravenous dexamethasone twice a day, 20 cc of bromhexine syrup three times daily, 1 gr intravenous ceftriaxone twice a day, and 750 mg oral levofloxacin once daily. During her hospital stay, the patient expectorated two thick white sputum samples, which were later found to contain hair upon microscopic evaluation (Figure 1). Due to anxiety and irritability, the patient underwent psychiatric consultation. The patient has a strained relationship with her father and displays a high level of dependence on her mother. Notably, she had a history of co‐sleeping with her mother until 4 years ago. In addition, the patient reported experiencing two 30‐minute‐long episodes of dyspnea, palpitations, feelings of choking, fear of dying, and paresthesia in her limbs over the past 6 months. Based on these symptoms, the patient was diagnosed with panic disorder and prescribed citalopram. Although the patient denied having any history of trichotillomania or trichophagia, she confirmed close contact with a pet dog. Her dyspnea and leukocytosis gradually improved, and the patient was discharged on the seventh day.

## DISCUSSION

The presence of hair in the respiratory tract is extremely uncommon and can be attributed to various causes such as trichobezoar, hair acting as a foreign body, or trichoptysis secondary to teratoma.^1^, ^4^, ^5^, ^6^ Few case reports have been documented on trichobezoars in the respiratory tract. In 2022, Adeli et al,^1^ described a 57‐year‐old woman with a history of trichotillomania and depression who presented with dyspnea, dysphagia, and hoarseness. A trichobezoar was incidentally found and removed from her hypopharynx, and a few strands of hair were also observed in her oesophagus. In another case reported by Suttithawil et al.,^4^ a 51‐year‐old woman with a Montgomery T tube experienced obstruction due to a trichobezoar. The presence of pet dogs was identified as a possible cause for trichobezoar formation in this case.

The presence of trichobezoars in the GI tract of humans was first reported in 1779.^1^ This condition is commonly observed among young females who experience trichotillomania. Adverse outcomes associated with bezoars consist of perforation, peritonitis, intestinal blockage, intussusception, and pancreatitis. The extent of complications largely depends on the location of the bezoar. Management strategies primarily depend on the size, location, and type of bezoar. Therapeutic options can include endoscopic removal, laparoscopic removal, and gastrotomy.^3^

In this case report, we present the clinical course of a young woman with underlying psychological issues who presented with hair in both her respiratory and gastrointestinal systems. The trichobezoar initially presented as a mucus plaque, but its true nature was later revealed. The location of the trichobezoar in the respiratory tract remains unclear, despite imaging not detecting it, so we use the term “respiratory bezoar” to refer to it.

The role of drug therapy, particularly dexamethasone and bromhexine, in contributing to the spontaneous expectoration of the respiratory bezoar should be considered. We cannot confirm whether the patient was accurate in denying trichotillomania and trichophagia. Her pet dog may have contributed to the formation of the trichobezoar; however, it is unlikely. Eosinophilia, which was observed in our patient, is a potential clinical indicator for trichobezoars. Additionally, hair filaments were found incidentally in the patient's gastrointestinal tract, and occult blood may indicate their presence.

In conclusion, this case report presents an uncommon occurrence of hair accumulation in both the respiratory and gastrointestinal tracts of a 25‐year‐old female patient. The clinical presentation involved worsening dyspnea, cough, epigastric pain, and leukocytosis. The therapeutic approach included drug therapy, which potentially facilitated the expectoration of the respiratory trichobezoar. The presence of eosinophilia and positive occult blood might serve as clinical indicators for trichobezoars in respiratory and GI tracts, respectively. Despite the patient's denial of trichotillomania and trichophagia history, it is reasonable to consider these habits as the cause of the formation of the trichobezoar. Overall, trichobezoars found in uncommon anatomical locations like the respiratory tract should be regarded as a potential diagnosis. Acquisition more knowledge about such instances can contribute to the timely diagnosis and proper treatment of these cases.

## AUTHOR CONTRIBUTIONS

Siavash Abedi involved in the collecting of sample and data. Masoud Aliyali, Amir‐Hassan Bordbari, and Mostafa Soleymani comprised in the interpretation writing, and editing of the manuscript. Zakaria Zakariaei and Mahdi Fakhar prepared the draft and submitted the manuscript. All authors reviewed and approved the final version of the manuscript.

## CONFLICT OF INTEREST STATEMENT

None declared.

## ETHICS STATEMENT

Written informed consent was obtained from the patient for publication of this case report.

The study was approved by our local ethics committee.

## Data Availability

The data are available with the correspondence author and can be achieved on request.
